# Effects of a Home-Based Physical Activity Program on Blood Biomarkers and Health-Related Quality of Life Indices in Saudi Arabian Type 2 Diabetes Mellitus Patients: A Randomized Controlled Trial

**DOI:** 10.3390/life13061413

**Published:** 2023-06-19

**Authors:** Jonathan Sinclair, Hussein Ageely, Mohamed Salih Mahfouz, Abdulrahman Ahmed Hummadi, Hussain Darraj, Yahia Solan, Robert Allan, Fatma Bahsan, Hassan AL Hafaf, Ali Abohadash, Mohammed Badedi, Lindsay Bottoms

**Affiliations:** 1 Research Centre for Applied Sport, Physical Activity and Performance, School of Sport & Health Sciences, Faculty of Allied Health and Wellbeing, University of Central Lancashire, Lancashire PR1 2HE, UK; rallan1@uclan.ac.uk; 2 Department of Family and Community Medicine, Faculty of Medicine, Jazan University, Jazan 45142, Saudi Arabia; hageely@jazanu.edu.sa (H.A.); mmahfouz@jazanu.edu.sa (M.S.M.); 3 Jazan Diabetes and Endocrinology Center, Jazan 45142, Saudi Arabiadr.h.d@hotmail.com (H.D.); y.solan@jazan.edu.sa (Y.S.);; 4 Jazan Health Affairs, Jazan 45142, Saudi Arabia; 5 Centre for Research in Psychology and Sport Sciences, School of Life and Medical Sciences, University of Hertfordshire, Hertfordshire AL10 9AB, UK; l.bottoms@herts.ac.uk

**Keywords:** hemoglobin A1c (HbA1c), Saudi Arabia, diabetes mellitus, exercise, physical activity

## Abstract

The purpose of this study was to undertake a randomized control trial examining the effects of a 12-week home-based physical activity program on Saudi Arabian adults with type 2 diabetes. Sixty-four patients with type 2 diabetes mellitus were recruited from the Jazan Diabetes and Endocrinology Center, located in the Jazan region of southwestern Saudi Arabia. Patients were randomly assigned to either control, i.e., usual care (males = 46.9% and females = 53.1%, age  =  45.88 ± 8.51 years, mass  =  76.30 ± 15.16 kg, stature  =  160.59 ± 8.94 cm, body mass index (BMI)  =  29.73 ± 6.24 kg/m^2^, years since diagnosis  =  8.12 ± 6.22 years) or a home-based physical activity (males = 50% and females = 50%, age  =  42.07 ± 9.72 years, mass  =  74.58 ± 13.67 kg, stature  =  158.94 ± 9.38 cm, BMI  =  29.44 ± 4.38 kg/m^2^, years since diagnosis  =  12.17 ± 8.38 years) trial arms. The home-based physical activity group was required to undertake aerobic training by increasing their habitual step count by 2000 steps per day and performing resistance training 3 times per week for 12 weeks. The primary outcome was hemoglobin A1c (HbA1c), and secondary measures of anthropometrics, blood biomarkers, physical fitness, and patient-reported quality of life outcomes pertinent to type 2 diabetes were measured at timepoints, i.e., baseline, 12 weeks, and 24 weeks (follow-up). Intention-to-treat analyses revealed no significant alterations in the primary outcome (control: baseline = 8.71%, 12-weeks = 8.35%, and follow-up = 8.72%; home-based physical activity: baseline = 8.32%, 12-weeks = 8.06%, and follow-up = 8.39%) between trial arms. However, improvements in psychological wellbeing at follow-up measured using the Patient Health Questionnaire-9 were significantly greater in the home-based physical activity group (baseline = 6.84, 12-weeks = 5.96, and follow-up = 5.00) compared to the control (baseline = 6.81, 12-weeks = 5.73, and follow-up = 8.53). No other statistically significant observations were observed. Home-based physical activity is not effective in mediating improvements in HbA1c levels or secondary hematological, blood pressure, anthropometric, or fitness indices. However, given the link between psychological wellbeing and the etiology/progression of disease activity in type 2 diabetes, home-based physical activity may be effective for tertiary disease management. Future trials should examine the efficacy of relative exercise intensities greater than those in the current study.

## 1. Introduction

Globally, type 2 diabetes represents a highly prevalent chronic disease that places a significant burden on life and healthcare costs [1]. In 2021, the International Diabetes Federation indicated that there were 537 million individuals with diabetes worldwide [2], representing a global prevalence of 10.5%, and concerningly, numbers are projected to rise to 783 million by 2045. Globally, over 90% of people with diabetes have type 2 diabetes [2]. As such, type 2 diabetes has been dubbed the 21st century’s primary global healthcare challenge by both the World Health Organization (WHO) and the International Diabetes Federation [2,3].

Type 2 diabetes is characterized by insensitivity to insulin, declining insulin production, and eventual pancreatic beta-cell failure [4], leading to reductions in glucose transport into the liver [1]. The condition is associated with a multifactorial etiology that comprises both genetic and modifiable lifestyle factors [5]. Lifestyle measures, including physical activity, are key factors for prevention and self-management in patients with type 2 diabetes [6]. Importantly, enhanced physical activity levels have long been considered a cornerstone for both type 2 diabetes prevention and management [7].

Owing to increasing levels of economic progression, urbanization, and negative lifestyle alterations, the highest prevalence of type 2 diabetes is in the Middle East and North Africa [8]. In the UK, the reported prevalence of type 2 diabetes is reported as being 5.26%, yet in Saudi Arabia, a recent review study reported that the prevalence is much higher, at around 33%, and it is expected to increase to 45.8% by 2030 [9]. The WHO has recently ranked Saudi Arabia as having the second highest rate of diabetes in the Middle East (7th highest in the world), with an estimated population of 7 million living with diabetes and more than 3 million with pre-diabetes [10]. Concerningly, recent studies from Saudi Arabia revealed that healthcare costs associated with diabetes have risen by 500% in the last 10 years [11] and that diabetes directly costs around 13.9% of the total health expenditure [12]. Based on expected population growth and the aforementioned increase in diabetes prevalence, it is expected that costs will triple by 2030 [13]. Therefore, it is essential that research designed to improve health/quality of life outcomes in type 2 diabetic patients be conducted in Saudi Arabia to decrease the social and personal costs of this disease.

Physical activity is a renowned health-enhancing modality [14]. According to the WHO, lack of physical activity is among the primary risk factors for non-communicable diseases and total mortality [15]. While a lack of physical activity accounts for 9% of premature deaths [16], it has been shown to significantly attenuate non-communicable diseases, including type 2 diabetes [17]. The WHO introduced a global initiative to attenuate inactivity levels [18]; however, concerningly, recent analyses show that such enterprises are operational in only 56% of WHO member countries [19]. During the past several decades, Saudi Arabia has witnessed significant economic and technological growth that has mediated negative lifestyle alterations [20]. The overall rate of physical inactivity among Saudi Arabian adults was 80.5% [14,15], making a lack of physical activity a major concern.

Clinical and epidemiological evidence has shown hemoglobin A1c (HbA1c) to be the gold standard for monitoring and chronic management of type 2 diabetes mellitus [21]. Physical activity significantly effects nutrient metabolism, meaning that regular exercise may improve glycemic control [22], as well as other health-related indices such as blood pressure, body adiposity, and lipid levels [23]. Randomized intervention analyses have shown that aerobic exercise is effective in improving HbA1c [22,24,25], insulin sensitivity [26,27,28], blood pressure [24], body mass index (BMI)/adiposity [22,29,30], fitness levels [22,31], and symptoms of peripheral neuropathy [32,33]. Furthermore, although traditionally aerobic exercise was considered the gold standard for the management of type 2 diabetes mellitus, in recent years there has been increased recognition of the independent benefits of resistance training. Randomized controlled trials have confirmed that standalone resistance training is able to mediate improvements in HbA1c [34,35,36,37,38], insulin sensitivity [38,39,40], blood pressure [35,37,38], BMI/adiposity [30,39], fitness levels [38], and fasting glucose [37,39,40]. Finally, combined resistance and aerobic training has been shown to offer a synergistic and incremental effect on HbA1c [30,36,38,41,42], insulin sensitivity [29,38], blood pressure [38], BMI/adiposity [29,30], blood lipids [38], fitness levels [30,38], and fasting glucose [41,42], indicating that a blended approach represents the most effective approach to the management of type 2 diabetes through the medium of physical activity.

Therefore, while previous trials have shown physical activity to be effective in improving clinical outcomes in type 2 diabetes, and it is abundantly clear that the Saudi Arabian population needs to become more physically active in order to control the rising incidence of non-communicable diseases, the process of enhancing engagement with physical activity remains a significant challenge. Possible barriers to physical activity in Saudi Arabia cited in the current literature are lack of time, high-density traffic, poor air quality, lack of suitable exercise places/sports facilities, lack of friends/social support, gender (i.e., being female), cultural barriers, low self-confidence, lack of time, and environmental factors (i.e., high temperatures outdoors) [43,44,45,46].

### 1.1. Rationale

Previous analyses have shown that physical activity can be undertaken at home [47] and that home-based activity interventions can be effective [48]. With public modesty being extremely important in Islamic countries, allied to a lack of suitable exercise locations, traffic, and poor air quality, this indicates that there are several significant barriers to physical activity engagement in Saudi Arabia. Therefore, a home-based physical activity program with a social component individualized for males and females may present an ideal solution to increase physical activity and exercise participation in Saudi Arabia. There is, however, no available information concerning the efficacy of a home-based exercise program for type 2 diabetic patients. Accounting for the incidence of type 2 diabetes, the rate of physical inactivity in Saudi Arabia, and the specific barriers to physical activity in this region, a home-based approach to delivering physical activity appears to be strongly warranted.

### 1.2. Aims

The purpose of this research study is to undertake a randomized control trial examining the effects of a 12-week home-based physical activity program on Saudi Arabian adults with type 2 diabetes. The primary objective of this randomized trial is to examine the influence of the physical activity program on blood HbA1c levels relative to controls. Its secondary objectives are to determine whether the intervention impacts other anthropometric, blood biomarker, physical fitness, and patient-reported quality of life outcomes pertinent to type 2 diabetes.

### 1.3. Hypotheses

In relation to the primary outcome, the physical activity intervention will mediate more substantial reductions in HbA1c levels compared to the control group. Furthermore, for the secondary outcomes of blood pressure, anthropometric indices, and physical fitness, these will improve as a function of the physical activity intervention compared to the control.

## 2. Materials and Methods

### 2.1. Study Design and Setting

The complete protocol, including the study setting, consort diagram, randomization, recruitment process, and estimation of sample size, has been published in detail [49]. This investigation represents a 24-week (in total) parallel randomized controlled trial, with participants randomized at the individual level to their designated trial arm. The study design is described according to the updated guidelines for reporting parallel group randomized trials [50]. The trial was undertaken at the Jazan Diabetes and Endocrinology Center, located in the Jazan region of southwestern Saudi Arabia. The population of Jazan is relatively homogeneous, with inhabitants habitually sharing the same language, ethnicity, and religion [51].

After screening for eligibility and enrollment, participants were individually randomized by a computer program (Random Allocation Software) to 12-weeks of either home-based physical activity or control group. Primary and secondary outcome variables, as described in detail below, were assessed at baseline (12 weeks and at follow-up (24 weeks)). In agreement with previous trials of type 2 diabetes management, the primary outcome measure was the between-group change in HbA1c [22,52]. Secondary outcome measures were between-group differences in anthropometrics, blood pressure, resting heart rate, blood biomarkers, physical fitness, and patient-reported outcome indices.

### 2.2. Inclusion and Exclusion Criteria

#### 2.2.1. Inclusion Criteria

Inclusion criteria were a clinically established diagnosis of type 2 diabetes for at least 12 months, being previously sedentary, being aged over 18 years, having primary physician clearance for participation in the study, and having full capacity to provide informed consent.

#### 2.2.2. Exclusion Criteria

Exclusion criteria were any cognitive impairment precluding consent or participation, pregnancy, additional medical conditions that prevented safe physical activity (e.g., severe arthritis or advanced heart failure), and enrollment in any other clinical trial designed to influence type 2 diabetes symptoms.

### 2.3. Sample Size

Power calculations were performed for the primary outcome variable, i.e., the between-group change in HbA1c. An a priori power analysis was undertaken with a significance level of 5% and 80% power based on previous HbA1c change scores over 12 weeks (of a longer duration trial) following a physical activity intervention in patients with type 2 diabetes. This showed that a total sample size of 64 was necessary to achieve α = 5% and β = 0.80 and detect a change of 0.73 between groups [22], with a projected standard deviation of 0.95 in each group, accounting for a loss to follow-up rate of 20%.

### 2.4. Ethical Approval

This study was granted ethical approval by the University of Jazan, Health Research Ethics Committee (REF: 2177) and formally and prospectively registered as a trial (NCT04937296). 

### 2.5. Participants and Recruitment

This study was conducted with patients attending the Jazan Diabetes and Endocrinology Center. Recruiting materials were placed within the Diabetes and Endocrinology Center using public patient bulletin boards. Participants were recruited between January 2022 and November 2022. Interested individuals were afforded the opportunity to contact the research team for further study information and to ask any questions associated with participation in the study. Participants were first invited to attend an eligibility, enrollment, and familiarization session at the diabetes center and supported with their travel costs throughout the course of this investigation. Written informed consent was obtained from those willing to take part, and all participants were advised to maintain their previous routine medications and diets. Furthermore, those in the control group were requested to maintain their present lifestyle until the end of the final “follow-up” data collection session.

### 2.6. Intervention

#### 2.6.1. Home Based Physical Activity

Participants assigned to the intervention group were required to perform resistance exercises 3 times a week on alternating days for 12 weeks. Each session began with a 5-min warm-up and ended with a 5-min cool-down. Exercises were performed with a TheraBand and targeted all of the major muscle groups (see Table 1). The exercises were the squat, lunge, press-up, cross-body reach, reverse fly, lateral raise, biceps curl, triceps extension, frontal raise, and bridge. During the first 4 weeks, the participants completed 2 sets (with a 2-min rest between sets) of 10–12 repetitions of each exercise, resting for 30 s between each TheraBand exercise. This progressed to 15 repetitions during weeks 4–8. Finally, in weeks 9–12, participants performed 3 sets of 10–15 repetitions. A research nurse supervised the first exercise session in week 1 to show participants all of the exercises, then the second exercise session of week 1 to ensure the participants were performing all of the exercises correctly. The final exercise sessions of weeks 4 and 8 were also supervised in order to determine whether participants could progress onto the next stage of the program. These supervised sessions were completed either remotely via a video call or in person at the diabetic center. To ensure that the intervention was delivered in a safe and homogeneous manner, the research nurses completed training and were provided with videos demonstrating the exercises with instructions on how to do them. 

In addition to the resistance exercises, participants in the home-based physical activity group also performed aerobic exercises. Participants were asked to download an Arabic smart phone steps application in order to record the number of steps on day 1 of the intervention (assuming that on this day, they completed their normal daily activities that they would do most days of the week). Participants who did not possess the required smartphone technology were provided with a pedometer. They were then asked to add 2000 steps to their daily steps, and this amount became their daily step target. Once they had reached this target on 4 out of 5 days, they were then asked to increase their step goal by 500 (see Table 2). Compliance was assessed by the research nurses, who rated each participant at the end of the 12-week period on a 10-point Likert scale (1 representing the lowest and 10 denoting the highest possible engagement), allowing a percentage compliance score to be calculated. In accordance with Santos et al. [53], we determined high compliance with the home-based physical activity intervention to be 70–90%.

The participants were assigned to a single-sex WhatsApp group of up to nine people, where they were reminded to go out and walk and were able to motivate one another. The WhatsApp groups were introduced as a lack of friends/social support has been cited as a barrier to physical activity engagement in Saudi Arabia [43,44,45,46]. All participants in the intervention group received their usual care as well as the standard lifestyle education program, which is individualized to their needs and offered by the Diabetic Center. The education program consists of an appointment with a consultant who provides information about changing their lifestyle and activity levels. Following this, participants attend two visits to the medical education and nutrition clinics where they are provided with information and support materials on physical activity. Immediately prior to and preceding each exercise session, participants took their own capillary blood samples by finger-prick using a disposable lancet after cleaning with a 70% ethanol wipe. Capillary glucose levels were obtained using a handheld analyzer in order to monitor blood glucose levels and prevent hypoglycemia. 

#### 2.6.2. Control Group

The control group did not receive any physical activity; however, they were randomized into groups of up to nine people and allocated to a WhatsApp group (to control for any social interaction). The participants received their usual care as well as the aforementioned lifestyle education program provided at the Diabetes and Endocrinology Center.

### 2.7. Data Collection

All measurements were made at the Jazan Diabetes and Endocrinology Center and undertaken in an identical manner on three occasions, i.e., baseline, 12 weeks, and follow-up. 

#### 2.7.1. Demographic and Health Information

In accordance with the consensus statement for the investigation of type 2 diabetic patients [52], age, years since diagnosis, sex, race, smoking status, marital status, number of children, diabetes treatment, blood pressure-lowering therapy, and educational level were obtained by self-report at baseline.

#### 2.7.2. Anthropometric Measurements

Anthropometric measures of body mass (kg) and stature (m) (without shoes) were obtained and used to calculate body mass index (BMI) (kg/m^2^). Stature was measured using a stadiometer, and mass was measured using standard weighing scales. Finally, waist circumference was measured at the midway point between the inferior margin of the last rib and the iliac crest and hip circumference around the pelvis at the point of maximum protrusion of the buttocks without compressing the soft tissues [54], allowing the waist-to-hip ratio to be quantified. Waist and hip circumference were obtained at 12-week follow-up. Anthropometric measures were obtained on three occasions, and the mean value was extracted for analysis.

#### 2.7.3. Blood Pressure

Blood pressure measurements were undertaken in an upright seated position. Peripheral measures of systolic and diastolic blood pressure as well as the resting heart rate were measured via a non-invasive, automated digital blood pressure monitor, adhering to the recommendations specified by the European Society of Hypertension [55], with the cuff positioned at the level of the heart and the back and arm supported. Three readings were undertaken, each separated by a period of 1 min [56], and the mean of the last 2 readings was used for analysis.

#### 2.7.4. Blood Biomarkers

Whole blood samples were collected from the antecubital vein into blood collection tubes coated with ethylenediaminetetraacetic acid (EDTA) for HbA1c and serum gel/clot activator tubes for fasting glucose and lipid profile analyses. The samples were analyzed immediately following blood collection. HbA1c was analyzed using a fully automated benchtop analyzer (Tosoh HLC-723G8, Tosoh Bioscience, Tokyo, Japan), and glucose and blood lipid profiles were quantified via a chemistry analyzer (VITROS XT 3400, Raritan, NJ, USA).

HbA1c (%), fasting glucose (mg/dL), and blood lipid profiles (triglycerides (mg/dL), total cholesterol (mg/dL), high density lipoprotein (HDL) cholesterol (mg/dL), and low density lipoprotein (LDL) cholesterol (mg/dL)) were extracted in accordance with the Nano et al. [52] consensus statement. Furthermore, the ratios between total and HDL cholesterol and between LDL and HDL cholesterol levels were also determined in accordance with Millán et al. [57]. Finally, the triglyceride glucose index (TyG index) was calculated using Equation (1) as the natural logarithm of the product of plasma glucose and triglycerides divided by two [58].

Ln (fasting triglycerides [mg/dL] × fasting plasma glucose [mg/dL]/2)(1)

#### 2.7.5. Physical Fitness

Physical activity is the primary intervention modality, and low cardiorespiratory fitness is significantly associated with impaired fasting glucose control and an independent predictor of all-cause mortality in type 2 diabetes [59]. Therefore, to assess the efficacy of the physical activity intervention itself, physical fitness itself was examined using the number of stages completed on the Chester Step test at the timepoints outlined above [60].

#### 2.7.6. Patient-Reported Outcome Measures

In further accordance with the consensus statement of Nano et al. [52], two generic and one diabetes-specific tools were utilized at baseline, after the 12-week intervention period, and at follow-up. The five-item WHO Well-Being Index (WHO-5), the Patient Health Questionnaire-9 (PHQ-9), and the Problem Areas in Diabetes (PAID) scale. In addition, as type 2 diabetes is associated with a higher incidence of sleep disorders [61], sleep quality was examined using the Pittsburgh Sleep Quality Index (PSQI) at baseline, after the 12-week intervention period, and at follow-up. The WHO-5 and PHQ-9 have been shown to be valid and reliable screening tools for depression and as outcome measures in clinical trials [62,63]. Similarly, the PAID scale has been shown to be a valid, reliable, and sensitive research tool in patients with diabetes [64], and the PSQI has excellent test–retest reliability, specificity, and validity in research and clinical settings [65]. All questionnaires were translated into Arabic.

### 2.8. Statistical Analysis

All continuous experimental data are presented as means and standard deviations (SD). A statistical analysis of all continuous demographic and health variables was undertaken to compare the two groups at baseline using linear mixed effects models, with group modeled as a fixed factor and random intercepts by participants. In addition, to compare categorical demographic and health information, Pearson chi-square tests of independence were utilized to undertake bivariate cross-tabulation comparisons between the two trial groups for non-continuous baseline variables. 

Furthermore, in order to contrast the magnitude of the changes at both 12-weeks and follow-up between the two trial groups, linear mixed effects models were employed, with group modeled as a fixed factor and random intercepts by participants adopted, adjusted for baseline values modeled as a continuous fixed covariate [66,67]. For linear mixed models, the mean difference (*b*), t-value, and 95% confidence intervals of the difference are presented. We undertook these analyses on an intention-to-treat basis and adopted the restricted maximum-likelihood method.

In accordance with Sinclair et al. [67], changes from baseline to 12-weeks and to follow-up were utilized to create binary variables, i.e., improve/no change. Pearson chi-square tests of independence were then used to undertake bivariate cross-tabulation comparisons between the two trial groups to test differences in the number of participants who exhibited improvements in each of the experimental measures, the number lost to follow-up, and the number of adverse outcomes in each group. Chi-squared probability values were calculated using Monte Carlo simulation. All analyses were conducted using SPSS v27 (IBM, SPSS), and statistical significance was accepted at the *p* ≤ 0.05 level.

## 3. Results

### 3.1. Demographic and Health Information

Comparisons in demographic and health indices between trial arms showed that significantly more patients in the control trial arm utilized Sitagliptin medication compared to the home-based physical activity group (*X*^2^ _(1)_ = 8.90, *p* = 0.003). All other comparisons were non-significant (*p* = 0.08–1.00) (Table 3).

### 3.2. Compliance, Loss to Follow Up, and Adverse Events

**Figure 1:**
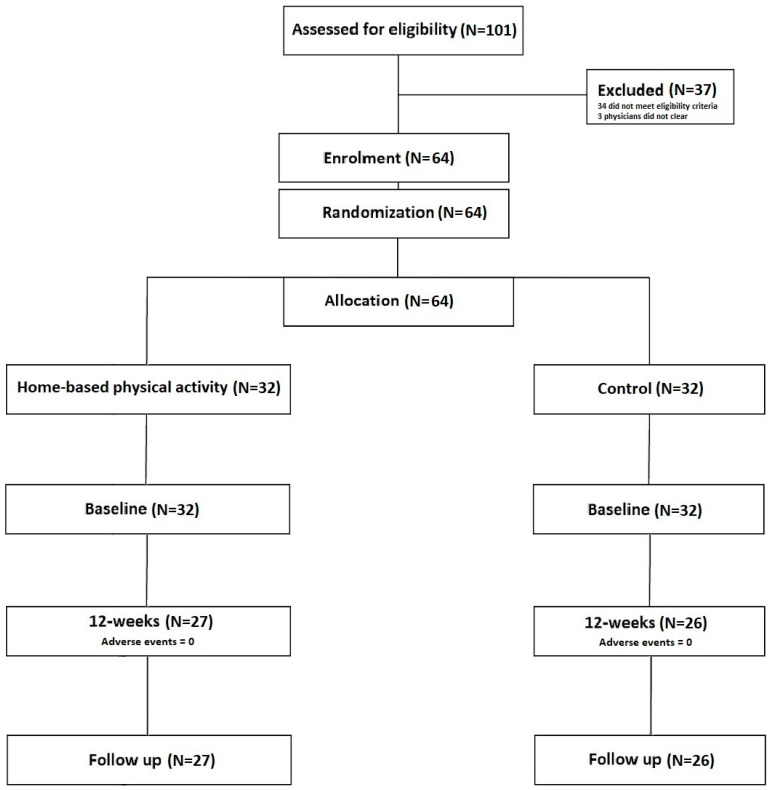
A consort diagram showing participant flow throughout the study.

Compliance in the home-based physical activity group was found to be 82.41 ± 18.21%. At 12 weeks, loss to follow-up in each group was control (18.75%) and home-based physical activity (15.63%), and the number of adverse effects was control (0%) and home-based physical activity (0%). Chi-square tests were non-significant (*X*^2^ _(1)_ = 0.17, *p* = 0.68) and *X*^2^ _(1)_ = 0.00, *p* = 1.00), indicating that there were no statistically significant differences between trial arms in either loss to follow-up or adverse events. At the follow-up timepoint, no further drops were evident (Figure 1).

**Table 3:** Baseline and general characteristics of the participants by the study arms.

	Total		Control		Home-Based Physical Activity	
	Mean	SD	Mean	SD	Mean	SD
Sex	Male = 48.4%		Male = 46.9%		Male = 50.0%	
	Female = 51.6%		Female = 53.1%		Female = 50.0%	
Age (yrs)	44.10	9.22	45.88	8.51	42.07	9.72
Mass (kg)	75.44	14.35	76.30	15.16	74.58	13.67
Stature (cm)	159.77	9.13	160.59	8.94	158.94	9.38
BMI (kg/m^2^)	29.59	5.35	29.73	6.24	29.44	4.38
Smoking status	Yes = 12.5%		Yes = 15.6%		Yes = 9.4%	
	No = 78.1%		No = 78.1%		No = 78.1%	
	Previous = 9.4%		Previous = 6.3%		Previous = 12.5%	
Marital status	Married = 84.4% Widowed = 4.7% Divorced = 3.1% Single = 7.8%		Married = 75.0% Widowed = 9.4% Divorced = 3.1 Single = 12.5%		Married = 93.8% Divorced = 3.1% Single = 3.1%	
Children	0 = 17.2% 1 = 3.1% 2 = 9.4% 3 = 14.1% 4 = 9.4% 5 = 9.4% 6+ = 37.5%		0 = 21.9% 2 = 9.4% 3 = 14.1% 4 = 9.4% 5 = 9.4% 6+ = 37.5%		0 = 12.5% 1 = 6.3% 2 = 9.4% 3 = 15.6% 4 = 12.5% 5 = 9.4% 6+ = 34.4%	
Years since diagnosis	9.77	7.38	8.12	6.22	12.17	8.38
Ethnicity	Arab = 98.4%, African = 1.6%		Arab = 96.9%, African = 3.1%		Arab = 100%	
Diabetes medication	Metformin = 81.3%		Metformin = 84.4%		Metformin = 78.1%,	
	Sitagliptin = 17.2%		Sitagliptin = 31.3%		Sitagliptin = 3.1%	
	Insulin = 43.8%,		Insulin = 56.3%		Insulin = 31.3%	
	Empagliflozin =9.4%		Empagliflozin =12.5%		Empagliflozin =6.3%	
Blood pressure lowering medication	Yes = 39.1%		Yes = 46.9%		Yes = 31.3%	
	No = 60.9%		No = 53.1%		No = 68.7%	
Education	Less than high school = 26.6%		Less than high school = 26.6%		Less than high school = 18.8%	
	High school = 20.3%		High school = 20.3%		High school = 21.9%	
	Associates degree = 20.3%		Associates degree = 20.3%		Associates degree = 25.0%	
	Bachelor’s degree = 31.3%		Bachelor’s degree = 31.3%		Bachelor’s degree = 31.3%	
	Doctoral = 1.6%		Doctoral = 1.6%		Doctoral = 3.1%	

### 3.3. Anthropometric Measurements

There were no significant differences between trial arms in the magnitude of the changes in waist circumference or waist-to-hip ratio at either of the experimental time points (*p* = 0.140–0.721) (Table 4). Chi-squared tests were also non-significant (*p* = 0264–0.950).

### 3.4. Blood Pressure

There were no significant differences between trial arms in the magnitude of the changes in systolic or diastolic blood pressure at either of the experimental time points (*p* = 0.537–0.813) (Table 4). Chi-squared tests were also non-significant (*p* = 0.09–0.535).

### 3.5. Physical Fitness

There were no significant differences between trial arms in the magnitude of the changes in the number of stages completed of the Chester step test at either of the experimental time points (*p* = 0.689–0.728) (Table 4). Chi-squared tests were also non-significant (*p* = 0.722–0.831).

### 3.6. Patient-Reported Outcome Measures

Improvements in PHQ-9 at the follow-up time point were significantly greater (*b* = 3.03 (95% CI = 0.46–5.60), t = 2.38, *p* = 0.022) in the home-based physical activity group. There were, however, no significant differences between trial arms in the magnitude of the changes in WHO-5, PHQ-9 (to 12-weeks), PAID, and PSQI scales at either of the experimental time points (*p* = 0.133–0.932) (Table 4). Chi-squared tests were also non-significant (*p* = 0.163–0.974).

### 3.7. Blood Biomarkers

There were no significant differences between trial arms in the magnitude of the changes in glucose, total cholesterol, LDL cholesterol, HDL cholesterol, triglycerides, HbA1c, TYG index, Total:HDL ratio, and LDL:HDL ratio at either of the experimental time points (*p* = 0.08–0.912) (Table 5). Chi-squared tests were also non-significant (*p* = 0.129–0.937).

## 4. Discussion

The current study aimed to investigate the influence of a home-based physical activity program on health-related indices pertinent to type 2 diabetes compared to control in Saudi Arabian adults. Owing to its unique amalgamation of social, economic, and environmental factors, it was envisioned that a home-based physical activity program may present an ideal solution to increase physical activity in Saudi Arabia. To date, this represents the first investigation to explore the effects of home-based physical activity using a randomized controlled trial in Saudi Arabia. The primary aim was to determine whether HbA1c levels were improved as a function of home-based physical activity, whereas the secondary aim(s) were to explore the effects of the intervention on other anthropometric, blood biomarker, physical fitness, and patient-reported quality of life outcomes pertinent to type 2 diabetes.

In relation to the primary outcome, the current investigation does not support our hypothesis in that there were no significant reductions in HbA1c levels in the home-based physical activity groups compared to placebo. This observation is not consistent with the observations of previous non-home-based randomized controlled trials using a combination of aerobic and resistance training [30,36,38,41,42]. However, this observation concurs with the feasibility findings from a previous trial utilizing a fully home-based physical activity program including both aerobic and resistance training modalities [68] and those from a trial utilizing only resistance training [69]. As the individuals included in this trial were previously sedentary, from the standpoint of patient safety, the exercises included in the home-based physical activity intervention were of low to moderate intensity, akin to activities of daily living. Therefore, taking into account the recent recommendations of Mustapa et al. [70] suggesting aerobic activity with a frequency of three to five times per week at moderate intensity, along with moderate intensity free and body weight resistance training exercises, is optimal to mediate improvements in type 2 diabetic patients. It can be speculated that the prescribed exercise regimen was not sufficiently intense to mediate improvements in HbA1c levels. Regardless, the observations from the current investigation indicate home-based physical activity, as specifically adopted in the current investigation, is not effective in mediating improvements in HbA1c levels in Saudi Arabian patients with type 2 diabetes.

The observations from this trial also did not reveal any significant improvements in secondary hematological, blood pressure, anthropometric, or fitness indices. These observations are once again inconsistent with the findings from previous non-home-based randomized interventions using amalgamized aerobic and resistance training approaches [29,30,38,41,42]. Furthermore, these findings oppose those of Plotnikoff et al. [69], who showed significant reductions in strength and insulin control following home-based resistance training, but do concur with the observations of Krousel-Wood et al. [68] using a combination of aerobic and resistance training. Once again, taking into account the recommendations of Mustapa et al. [70], it can be speculated that the physical activity utilized in this trial was not of sufficient intensity to mediate improvements in hematological, blood pressure, anthropometric, or fitness indices. The current investigation nonetheless suggests that the home-based physical activity program adopted in the current investigation was ineffective in facilitating improved hematological, blood pressure, anthropometric, or fitness parameters in Saudi Arabian type 2 diabetes patients.

However, although this trial did not reveal any significant improvements in haematological, blood pressure, anthropometric, or fitness indices compared to control, in agreement with the home-based trial of Plotnikoff et al. [69], the current investigation importantly revealed that this condition was able to mediate statistical improvements in psychological wellbeing at follow-up via the PHQ-9 scale (Table 4). Type 2 diabetes mellitus is associated with increased levels of depression/anxiety [71], and the presence of diminished psychological wellbeing exacerbates disease prognosis, increases medical therapy non-compliance [72], decreases the quality of life [73], and increases mortality [74]. Therefore, taking into account the importance of psychological wellbeing to the etiology and progression of disease activity and the aforementioned improvements noted in the home-based physical activity group, this observation is clinically and practically meaningful. Although the exact physical/biological mechanism responsible for the improvements in psychological wellbeing mediated by physical activity is not currently known, several conceivable mechanisms exist that may explain our findings, including thermogenic, endorphin, monoamine, distraction, and self-efficacy effects [75]. Regardless, the observations from the current trial suggest that home-based physical activity may represent a useful mechanism for tertiary disease management, pertinent to the etiology and progression of disease activity in type 2 diabetes.

Overall, the current trial demonstrated a very low number of adverse events, a good level of compliance, and a high retention rate in the intervention group. Therefore, it can be concluded that home-based physical activity is a safe and tolerable modality. This trial showed that no significant improvements in HbA1c levels or secondary hematological, blood pressure, anthropometric, or physical fitness indices were evident in the home-based physical activity group. However, significant improvements in psychological wellbeing were also observed in this trial arm. The lack of statistical improvements in the primary and pertinent secondary outcomes in the home-based physical activity group may be attributable to a lack of sufficient physical activity intensity. Therefore, future analyses seeking to mediate biological as well as psychological improvements should seek to examine the clinical efficacy of relative exercise intensities above and beyond those investigated in the current study. 

As with all experimental research, this trial is not without limitations. Firstly, the fact that nutritional intake was not standardized or quantified as part of the current trial may serve as a potential drawback. Previous analyses have shown that dietary patterns as well as physical activity have an important role to play in the etiology and management of type 2 diabetes [76]. Examination of nutritional intake during the intervention period could have provided additional information regarding the mechanisms responsible for some of the observations in this investigation, such as the increased waist circumference noted in each trial arm. Therefore, despite the inherent challenges associated with the accurate quantification of dietary intake [77], future analyses examining the effects of physical activity in type 2 diabetes should nonetheless seek to examine nutritional approaches during the intervention period. Furthermore, while the current investigation observed positive effects of home-based physical activity on psychological wellbeing indices, the mechanistic basis for these improvements remains unelucidated [75]. Therefore, given the link between depression and the etiology/progression of disease activity, future investigations should seek to explore and better exploit the mechanistic pathways of physical activity in order to improve disease prognosis in type 2 diabetes patients. Furthermore, the fact that participants were randomized into their designated trial arms without consideration for their previous levels of physical activity, medication, disease activity, or other experimental indices may serve as a potential drawback. Therefore, while comparative analyses at baseline showed only extremely small differences between groups in pharmaceutical management, future analyses may seek to adopt a stratified random sampling approach when exploring the effects of physical activity on indices pertinent to the etiology and management of this condition. 

## 5. Conclusions

The current study aimed to investigate, using a randomized controlled trial, the influence of a home-based physical activity program on HbA1c and other health-related indices pertinent to type 2 diabetes mellitus in Saudi Arabian adults. This trial notably showed no significant improvements in HbA1c levels or secondary hematological, blood pressure, anthropometric, or physical fitness indices in the home-based physical activity group. Suggesting that home based physical activity, as specifically adopted in the current investigation, is not effective in mediating improvements in these outcomes. However, this study did, importantly, show improvements in psychological wellbeing with home-based activity compared to control. Given the link between depression and the etiology/progression of disease activity, the current investigation indicates that home-based physical activity could represent a useful mechanism for tertiary disease management. Future intervention trials should consider examining the clinical efficacy of relative exercise intensities above and beyond those investigated in the current study.

## Figures and Tables

**Table 1:** Resistance exercises.

Stage	Resistance Exercises	Duration	Equipment
1 (weeks 1–4)	Squat Lunge Press-Up Cross Body Reach Reverse Fly Lateral Raise Biceps Curl Triceps Extension Frontal Raise Bridge	Rest for 30′s between each type of TheraBand exercise. Rest for 2 min before repeating the stage again. Sets: 2, Repetitions: 10–12	Latex free resistance band
2 (weeks 5–8)		Rest for 30 s between each type of TheraBand exercise. Rest for 2 min before repeating the stage again. Sets: 2, Repetitions: 15	Latex free resistance band
3 (weeks 9–12)		Rest for 30 s between each type of TheraBand exercise. Rest for 2 min before repeating the stage again. Sets: 3, Repetitions: 10–15	Latex free resistance band

**Table 2:** Training program.

Sunday	Monday	Tuesday	Wednesday	Thursday	Friday	Saturday
		Resistance Exercise		Resistance Exercise		
Walking (+2500 over daily step count)	Walking (+2500 over daily step count)	Walking (+2500 over daily step count)	Walking (+2500 over daily step count)	Walking (+2500 over daily step count)	Walking (+2500 over daily step count)	Walking (+2500 over daily step count)

**Table 4:** Anthropometric, blood pressure, physical fitness, and patient-reported outcome measurements as a function of each trial arm.

	Control						Home Based Physical Activity					
	Baseline		12-Weeks		Follow-Up		Baseline		12-Weeks		Follow-Up	
	Mean	SD	Mean	SD	Mean	SD	Mean	SD	Mean	SD	Mean	SD
Waist circumference (cm)	94.24	19.33	97.22	13.63	104.60	20.31	99.93	8.40	100.65	10.03	101.04	10.71
Waist-to-hip ratio	0.99	0.11	1.00	0.11	1.03	0.17	0.98	0.06	0.98	0.06	0.98	0.07
Systolic blood pressure (mmHg)	132.87	14.76	131.58	9.34	131.73	8.51	121.80	15.61	124.11	15.76	129.56	13.78
Diastolic blood pressure (mmHg)	80.27	9.83	78.50	10.31	79.13	7.75	82.72	13.32	78.37	9.46	81.56	11.34
Chester step test (Stages)	2.71	0.73	3.29	1.16	3.33	0.62	3.00	0.77	3.28	1.28	3.54	0.71
WHO-5	72.39	21.23	70.77	21.76	74.93	27.19	68.75	17.64	72.74	19.12	78.31	16.94
PHQ-9	6.81	5.10	5.73	5.14	8.53	4.37	6.84	2.71	5.96	3.48	5.00	3.53
PAID	22.10	15.56	21.49	19.21	15.83	14.11	21.33	13.70	19.26	20.06	17.12	19.29
PSQI	9.10	3.08	7.85	2.98	9.93	3.63	10.56	4.10	9.56	3.36	8.22	3.79

**Table 5:** Blood biomarker outcome measurements as a function of each trial arm.

	Control						Home Based Physical Activity					
	Baseline		12-Weeks		Follow-Up		Baseline		12-Weeks		Follow-Up	
	Mean	SD	Mean	SD	Mean	SD	Mean	SD	Mean	SD	Mean	SD
Glucose (mg/dL)	181.63	111.44	151.00	54.93	155.07	97.19	157.47	69.68	151.93	61.11	162.36	61.97
LDL cholesterol (mg/dL)	145.59	43.10	130.08	35.38	130.00	28.14	152.69	45.72	122.29	43.79	129.29	40.38
HDL cholesterol (mg/dL)	55.17	31.56	46.42	13.67	47.73	9.86	57.03	15.66	49.14	11.71	54.96	15.36
Total cholesterol (mg/dL)	228.43	46.75	204.65	39.52	204.47	27.03	247.84	51.36	195.00	51.51	207.07	48.04
Triglycerides (mg/dL)	146.10	84.23	142.85	90.05	160.48	61.19	129.32	59.15	117.64	57.80	144.06	70.72
HbA1c (%)	8.71	1.39	8.35	1.32	8.72	1.79	8.32	1.76	8.06	1.81	8.39	1.85
TyG index	9.25	0.75	9.12	0.57	9.26	0.68	9.05	0.66	8.93	0.69	9.20	0.61
Total cholesterol:HDL cholesterol ratio	4.68	1.50	4.72	1.53	4.43	0.99	4.64	1.48	4.14	1.28	3.96	1.26
LDL cholesterol:HDL cholesterol ratio	3.03	1.21	2.93	1.07	2.81	0.74	2.92	1.26	2.63	1.09	2.55	1.06

## Data Availability

The data presented in this study are available on request from the corresponding author.
